# Therapeutic Plateletpheresis in Patients With Thrombocytosis: Gender, Hemoglobin Before Apheresis Significantly Affect Collection Efficiency

**DOI:** 10.3389/fmed.2021.762419

**Published:** 2021-12-24

**Authors:** Hongqiang Jiang, Yanxia Jin, Yufeng Shang, Guolin Yuan, Dandan Liu, Jianfang Li, Cong Wang, Lu Ding, Xiqin Tong, Shishang Guo, Fayun Gong, Fuling Zhou

**Affiliations:** ^1^Department of Hematology, Zhongnan Hospital of Wuhan University, Wuhan, China; ^2^College of Life Sciences, Hubei Normal University, Huangshi, China; ^3^Department of Hematology, Xiangyang Central Hospital, The Affiliated Hospital of Hubei University of Arts and Science, Xiangyang, China; ^4^School of Physics and Technology and Key Laboratory of Artificial Micro- and Nano-structure of Ministry of Education, Wuhan University, Wuhan, China; ^5^School of Mechanical Engineering, Hubei University of Technology, Wuhan, China

**Keywords:** thrombocytosis, myeloproliferative neoplasms, platelet, therapeutic apheresis, collection efficiency

## Abstract

**Background:** Thrombocytosis is a common symptom in myeloproliferative neoplasms (MPN), and excessive proliferation may deteriorate into thrombosis, bleeding, myelofibrosis, and may ultimately convert to acute leukemia. This study aimed to investigate the collection efficiency of plateletpheresis (CEPP) and factors influencing its efficacy in patients with thrombocytosis.

**Materials and Methods:** From September 2010 to December 2016, 81 patients from two institutions in China with myeloproliferative neoplasms and thrombocytosis accompanied by severe symptoms were treated with plateletpheresis by Fresenius COM. TEC machine.

**Results:** After apheresis, the median CEPP was 20.71% (IQR: 9.99–36.69%) and median PLT reduction rate was 25.87% (IQR: 21.78–36.23%). Further analysis showed that no significant difference was observed between PLT count with 800–1,000 × 10^9^/L and > 1,000 × 10^9^/L. The PLT counts significantly decreased (*P* < 0.001) after plateletpheresis, the red blood cell (RBC), white blood cell (WBC), hemoglobin (HGB), and hematocrit (HCT) levels showed no significant differences before- or after- plateletpheresis. Multivariate analysis showed that female sex (*P* = 0.009) and HGB (*P* = 0.010) before apheresis were associated with CEPP. Female (*P* = 0.022), HCT (*P* = 0.001) and blood volume (*P* = 0.015) were associated with the PLT reduction rate. Furthermore, symptoms were relieved after apheresis in patients whose PLT count was 800–1,000 × 10^9^/L accompanied with symptoms.

**Conclusions:** It is reasonable to perform plateletpheresis when the PLT count is over 800 × 10^9^/L and patients are complicated by clinical symptoms such as dizziness, headache, somnolence, and stupor. Plateletpheresis is effective in removing PLTs especially in females with high HGB.

## Introduction

Myeloproliferative neoplasms (MPN) are a group of malignant proliferative disease of the bone marrow that originate from pluripotent hematopoietic stem cells, and include disorders such as polycythemia vera (PV) (1), essential thrombocythemia (ET) (2, 3), and primary myelofibrosis (PMF) (4). Excessive proliferation of myeloid cells in MPN may deteriorate into thrombosis, bleeding, myelofibrosis, and may finally convert to acute leukemia (5–8). Thrombocytosis is defined by platelet (PLT) counts >450 × 10^9^/L on blood routine examination (9), and abnormal megakaryocytopoiesis associated with elevated thrombopoietin (TPO) in the plasma leading to an increase in PLT count (10, 11). Dysregulated megakaryocytopoiesis has been associated with a driver mutation in one of three genes, JAK2, MPL, and CALR, which leads to constitutive and hyperactive JAK-STAT signaling and ultimately resulting in dysregulated cellular proliferation (12, 13). Excessive PLT counts usually causes various symptoms, such as ischemia, bleeding, headache, dizziness, and stroke (14, 15), and other subjective neurological symptoms (16). Bleeding and thrombosis are major causes of morbidity and mortality in patients with thrombocytosis (17, 18). Hydroxyurea or anagrelide and low-doses of aspirin can be used to treat thrombocytosis, but headache, palpitations, diarrhea, nausea, and other side-effects often occur (19).

Therapeutic apheresis is a relatively safe and useful therapy (20), which can be used as a rapid and short-term adjunct to conventional chemotherapy or immunotherapy (21). Therapeutic cytapheresis is used to remove excessive PLT or WBC (14). Plateletpheresis is one of the most important therapeutic strategies to decrease PLT count and treat thrombocytosis for patients with clinical manifestations including inflammation (22), hemorrhage (23), giddiness (24), headache (25), vertigo (26), and malaise (27), and to prevent ischemia or stroke (28). Beenu et al. (29) reported a case of chronic myeloid leukemia (CML) with thrombocytosis, whose PLT count decreased from 1,553 × 10^9^/L to 513 × 10^9^/L after two therapeutic plateletpheresis procedures. According to the 2019 American Society for Apheresis (ASFA) guidelines, thrombocytapheresis is used only as a bridging therapy along with cytoreduction therapy (9).

In this study, we analyzed the collection efficiency of plateletpheresis (CEPP) and its influencing factors in 81 MPN patients with thrombocytosis who underwent plateletpheresis. Patients with PLT count above 800 × 10^9^/L with obvious symptoms and PLT counts above 1,000 × 10^9^/L with or without symptoms were enrolled in this analysis.

## Patients and Methods

### Patients

From September 2010 to December 2016, 81 patients with MPN and thrombocytosis from Zhongnan Hospital of Wuhan University and Xiangyang Central Hospital were included in the analysis. The study was conducted in accordance with the Declaration of Helsinki and was approved by the Research Ethics Board of Zhongnan Hospital of Wuhan University and Xiangyang Central Hospital. Patients provided informed consent and data were collected from the electronic patient record. All patients were diagnosed with the World Health Organization (WHO) criteria for hematological diseases (3, 7). The decision of whether to perform plateletpheresis is individualized based on the patient's characteristics and physician preferences. Patients with PLT counts above 800 × 10^9^/L complicated with angina or other ischemic events, dizziness, headache, somnolence, or stupor, they should undergo plateletpheresis. We only conduct plateletpheresis for patients with acute symptoms, as outlined in the Methods section. Most patients only underwent a single apheresis, and only a second apheresis was performed when the number of platelets was very high and the symptom relief was not obvious. No complications appeared during the procedure. No other treatment modalities were used for these patients when plateletpheresis procedures were performed. None of the patients received chemotherapy until PLT counts decreased to <800 × 10^9^/L after apheresis followed by oral hydroxyurea (27, 29).

### Procedure and Operational Variables of Therapeutic Plateletpheresis

The Fresenius COM.TEC device (Fresenius Kabi, Germany) runs with a continuous blood flow (version: 04.03.07). Procedures were conducted by trained apheresis technicians and assisted by a professional hematologist. PLT collection by the COM.TEC cell separator was controlled *via* the PLT_5d protocol and C5L. At the beginning of plateletpheresis procedure, the donor's sex, age, body weight, height, PLT counts, pre-apheresis HCT, and blood products were entered into the device. The COM.TEC instrument parameters were set as follows: the whole blood flow rate set at 50 ml/min, processed blood volume was 6,000–8,000 ml, anticoagulant citrate dextrose-formula A (ACD-A) / whole blood ratio = 1:7.5. Replacement fluid was primarily ACD with some saline supplementation in the plasma constituent due to volume shifts induced by the removal of plasma in the discarded product. Prophylactic calcium, 500 ml normal saline, plus 40 ml 10% calcium gluconate intravenous infusion was administered to all patients to prevent hypocalcemia occurrence during apheresis. Plateletpheresis kits with 16 G-18 G blood collection puncture needle were used according to the manufacturer's instructions. Blood volume processed (BVP) was defined as the processed volume of blood circulation in the machine of the individual. For all procedures, the separation time, BVP, and collection capacity were recorded in detail.

### Evaluation of the Plateletpheresis Efficiency

Peripheral blood samples [2 ml in ethylene diamine tetra acetate (EDTA) as the anticoagulant] were collected from each patient (inlet line) before and 3 h after completing the plateletpheresis. Samples of plateletpheresis products were obtained for laboratory analysis. Complete blood count, blood biochemistry, immunoglobulin, and coagulation function were assessed pre- and post-apheresis after the first procedure. The CEPP and rate of platelet reduction were calculated as reported previously (30, 31):


$$\begin{array}{l} \;CEPP\left(\%\right)=total\;PLT\;yield\;\left(10^{11}\right)\;in\;collected\;product\;\times \;100\%\\ \;÷\frac{\left(PLT\;count\;before\;apheresis+\;PLT\;count\;after\;apheresis\right)\;\times \;blood\;volume\;processed}{2}\\ \;Rate\;of\;platelet\;reduction\;\left(\%\right)\\ =\frac{platelet\;count\;before\;apheresis-platelet\;count\;after\;apheresis}{platelet\;count\;before\;apheresis}\times 100\% \end{array}$$

### Statistics Analysis

Statistical analysis was performed using IBM SPSS 24.0. Continuous variable was estimated by normality test and the variances equal test. Chi-square test was used for categorical variable and Mann-Whitney-Wilcoxon test were used for non-parametric test. Paired Wilcoxon test was used to determine the statistical differences before and after plateletpheresis. Multivariate analysis was performed with a regression model using the step-by-step method. All *P*-values were two-sided, and *P* < 0.05 were considered significant.

## Results

### Clinical Characteristics of Patients With Thrombocytosis

The study included 81 MPN patients with thrombocytosis. There were 67 patients with essential thrombocythemia (ET), two patients with primary myelofibrosis (PMF), one patient with polycythemia vera (PV), five patients with CML, and two patients with myelodysplastic syndrome (MDS). Some of patients in our study were not new cases. For instance, the five CML patients included in our study were not newly diagnosed. They had been diagnosed as CML according to WHO diagnostic criteria and were hospitalized because of subjective symptoms such as dizziness and fatigue. The clinical characteristics and laboratory findings of 81 cases grouped by sex before plateletpheresis are summarized in Table 1. No significant difference of those indicates between the two groups was evident.

**Table 1:** Baseline information and laboratory findings pre-apheresis of 81 cases with thrombocytosis grouped by SEX.

**Characteristics**	**Female (*n* = 40)**	**Male (*n* = 41)**	***p*-value**
	**Number or Median**	**Number or Median**	
	**(Percentage or IQR)**	**(Percentage or IQR)**	
**General information**			
***Disease profile***			0.097
ET	35 (87.5)	32 (78.0)	0.379
PMF	2 (5.0)	0 (0)	0.241
PV	0 (0)	1 (2.4)	0.999
CML	3 (7.5)	2 (4.9)	0.675
MDS	0 (0)	2 (4.9)	0.494
MDS/MPN-U	0 (0)	4 (9.8)	0.130
Age (year)	63.0 (56.0–74.0)	62.00 (56.00–68.00)	0.361
**Laboratory findings**			
***Complete blood count***			
RBC (×10^12^/L)	3.53 (3.15–4.01)	3.41 (3.14–3.97)	0.548
WBC (×10^9^/L)	10.86 (7.94–14.53)	10.07 (7.85–15.32)	0.992
PLT (×10^9^/L)	1097.0 (951.5–1184.5)	1100.0 (890.0–1254.0)	0.817
MPV (fl)	8.25 (7.50–9.35)	8.80 (7.85–9.15)	0.604
MO (×10^9^/L)	0.59 (0.19–1.58)	1.08 (0.31–1.59)	0.283
LY (×10^9^/L)	1.90 (1.20–2.43)	1.94 (1.25–2.56)	0.521
NEU (×10^9^/L)	7.15 (5.57–9.72)	9.02 (4.90–10.07)	0.891
HCT (%)	31.00 (25.25–34.00)	31.00 (29.00–35.00)	0.182
HGB (g/L)	99.00 (86.25–114.00)	100.00 (92.50–125.00)	0.301
***Hepatorenal function***			
AST (u/L)	26.00 (20.00–38.00)	28.00 (17.50–38.00)	0.305
ALT (u/L)	20.00 (13.00–30.00)	22.00 (18.75–30.50)	0.259
TBIL (μmol/L)	15.05 (8.98–18.18)	17.70 (10.33–20.90)	0.339
BUN (mmol/L)	6.01 (4.58–7.33)	6.10 (4.60–7.22)	0.921
CREA (μmol/L)	68.20 (53.78–81.00)	69.20 (55.50–81.00)	0.992
UA (μmol/L)	333.00 (192.90–402.30)	286.00 (130.00–383.50)	0.191
***Electrolyte***			
K (mmol/L)	4.44 (3.90–5.05)	4.06 (3.63–4.98)	0.204
Na (mmol/L)	140.90 (139.00–143.25)	139.00 (137.00–142.00)	0.114
Cl (mmol/L)	105.00 (102.50–106.10)	105.00 (102.00–106.00)	0.946
Ca (mmol/L)	2.33 (2.10–2.48)	2.20 (2.10–2.44)	0.444
P (mmol/L)	1.20 (1.12–1.55)	1.15 (1.09–1.67)	0.411
***Serum proteins***			
TP (g/L)	69.40 (66.90–72.13)	68.30 (61.83–76.00)	0.596
ALB (g/L)	44.20 (39.30–49.25)	43.00 (38.85–52.00)	0.868
GLB (g/L)	26.00 (21.53–28.70)	25.00 (20.00–30.00)	0.751

*Chi-square test and Mann-Whitney-Wilcoxon test were used.*

*CML, chronic myeloid leukemia; ET, essential thrombocythemia; IQR, interquartile range; MDS, myelodysplastic syndrome; MDS/MPN-U, myelodysplastic/myeloproliferative neoplasm unclassifiable; PMF, primary myelofibrosis; PV, polycythemia vera*.

### Outcomes and Changes of Complete Blood Count and Other Biochemical Characters Before and After Plateletpheresis

Symptoms (such as dizziness, headache, somnolence, and stupor) of each patient whose PLT count was 800–1,000 × 10^9^/L improved after the apheresis sessions. The paired Wilcoxon test suggested that PLT count (*P* < 0.001) was significantly decreased after plateletpheresis (Table 2). There was no statistical difference in red blood cell (RBC) counts (*P* = 0.376), or hemoglobin (HGB) (*P* = 0.931) before or after plateletpheresis. The levels of hepatic and renal function indicators such as Blood Urea Nitrogen (*P* < 0.001), creatinine (*P* = 0.004), and uric acid (*P* < 0.001) decreased. The median serum calcium level was 2.30 mmol/L before plateletpheresis and 2.11 mmol/L after plateletpheresis (*P* = 0.001), and serum sodium slightly decreased after plateletpheresis (*P* = 0.026). The median of total protein (TP) was 69.00 g/L before plateletpheresis and 64.00 g/L after plateletpheresis (*P* < 0.001), albumin (ALB) was 43.00 g/L before plateletpheresis and 43.25 g/L after plateletpheresis (*P* < 0.001), and globulin (GLB) was 25.40 g/L before plateletpheresis and 23.00 g/L after plateletpheresis (*P* = 0.003) (Table 2). Overall, the values of hematologic indexes after plateletpheresis were still within the normal range. The results of coagulation function pre-apheresis and post-apheresis are shown in Table 2, and except APTT, the coagulation parameters had no statistical differences pre-apheresis and post-apheresis. In summary, when clearing PLT, there was little impact on the overall equilibrium in the body.

**Table 2:** The change of laboratory findings between pre- apheresis and post-apheresis.

**Laboratory**	**Pre-apheresis**	**Post-apheresis**	***p*-value**
**tests**	**Median (IQR)**	**Median (IQR)**	
***Complete blood count***			
RBC (×10^12^/L)	3.51 (3.18–4.00)	3.56 (3.20–3.91)	0.376
WBC (×10^9^/L)	10.07 (7.85–14.66)	9.36 (6.42–14.53)	<0.001
PLT (×10^9^/L)	1100.00 (917.50–1236.50)	782.00 (652.50–906.50)	<0.001
MPV (fL)	8.40 (7.75–9.20)	8.40 (7.55–9.35)	0.656
MO (×10^9^/L)	0.83 (0.26–1.57)	0.61 (0.24–1.29)	<0.001
LY (×10^9^/L)	1.92 (1.21–2.50)	1.81 (1.00–2.62)	0.003
NEU (×10^9^/L)	7.90 (5.20–9.78)	6.21 (4.67–10.35)	0.109
HCT (%)	31.00 (27.00–34.55)	31.00 (27.00–33.00)	0.008
HGB (g/L)	100.00 (91.00–117.90)	101.30 (94.50–119.50)	0.931
***Hepatorenal function***			
AST (u/L)	27.00 (20.00–38.00)	21.50 (16.00–33.25)	0.016
ALT (u/L)	21.00 (17.50–30.00)	20.00 (16.00–34.00)	0.532
TBIL (μmol/L)	16.90 (9.10–19.08)	15.00 (8.75–19.00)	0.713
BUN (mmol/L)	6.10 (4.60–7.25)	5.31 (4.50–6.30)	<0.001
CREA (μmol/L)	68.30 (54.05–81.00)	64.90 (54.00–75.15)	0.004
UA (μmol/L)	315.50 (144.75–402.30)	213.50 (113.00–316.25)	<0.001
***Electrolyte***			
K (mmol/L)	4.30 (3.74–4.99)	4.06 (3.73–4.88)	0.080
Na (mmol/L)	140.80 (137.90–143.00)	140.00 (137.00–142.00)	0.026
Cl (mmol/L)	105.00 (102.00–106.00)	104.00 (99.00–107.00)	0.415
Ca (mmol/L)	2.30 (2.10–2.45)	2.11 (1.97–2.35)	0.001
P (mmol/L)	1.17 (1.10–1.55)	1.11 (0.92–1.40)	0.037
***Serum proteins***			
TP (g/L)	69.00 (62.33–75.00)	64.00 (58.75–71.25)	<0.001
ALB (g/L)	43.00 (39.00–50.75)	43.25 (36.30–50.00)	<0.001
GLB (g/L)	25.40 (21.00–29.00)	23.000 (20.00–27.80)	0.003
***Coagulation factors***			
PT (s)	13.10 (11.50–14.20)	11.15 (10.70–13.05)	0.056
INR	1.10 (0.90–1.20)	1.02 (0.98–1.19)	0.492
PTTA (%)	90.00 (76.00–105.00)	100.00 (80.00–110.00)	0.131
APTT (s)	35.10 (28.90–40.00)	33.50 (29.10–34.90)	0.026
Fib-c (g/l)	325.00 (287.00–437.00)	285.00 (283.00–315.75)	0.112
FDP (mg/l)	3.00 (1.82–5.00)	16.82 (4.45–16.82)	0.713
DD (mg/l)	180.00 (146.75–550.00)	246.50 (121.25–1838.00)	0.715

*Paired Wilcoxon test was used.*

*IQR, interquartile range; PT, prothrombin time; INR, International Normalized Ratio; PTTA, Prothrombin time activity; APTT, activated partial thromboplastin time; Fib-c, fibrinogen-c; FDP, fibrinogen degradation products; DD, D-Dimer*.

### Product Data and Collection Efficiency of Plateletpheresis

**Figure 1:**
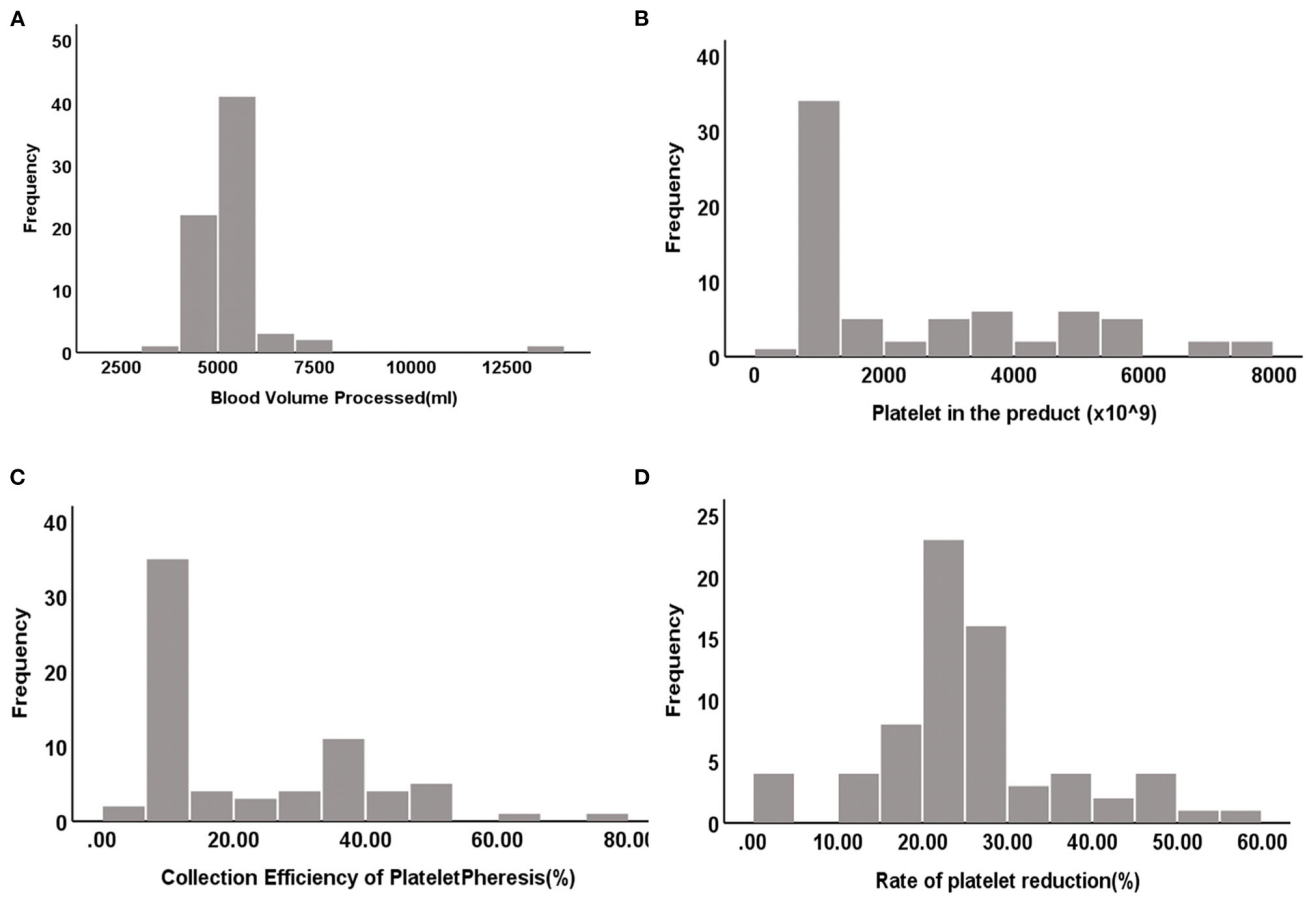
The distributed histograms of the BVP, PLT in the product, CEPP and Rate of platelet reduction. **(A)** Blood volume processed (BVP). **(B)** Platelet in the product. **(C)** CEPP. **(D)** Rate of platelet reduction.

The distribution histograms of the blood volume processed (BVP), PLTs in the product, CEPP, and Rate of platelet reduction are shown in Figure 1. The median PLT yield was 1328.5 × 10^9^/L [Interquartile range (IQR), 1105.75–3903.75 × 10^9^/L]. The median CEPP was 20.71% (IQR: 9.99–36.69%) and median PLT reduction rate was 25.87% (IQR: 21.78–36.23%). A sub-analysis with CEPP and platelet reduction is relevant comparing the outcome of patients with ET with the other patients. The median CEPP of patients with ET was 19.03%, and the CEPP is 24.34% in other patients (*P* = 0.235). Further, we also analyzed the effects of PLT count on the CEPP in this cohort. The PLT count was divided into two groups: 800–1,000 × 10^9^/L and >1,000 × 10^9^/L. There was no significant differences between PLT counts of 800–1,000 × 10^9^/L and >1000 × 10^9^/L (Table 3).

**Table 3:** The CEPP and PLT reduction rate of different PLT count groups in the current cohort.

**PLT count groups (×10^**9**^/L)**	**Number**	**CEPP (%) and IQR**	***P-*value**	**PLT reduction rate (%) and IQR**	***P-*value**
800–1,000	30	22.69 (10.33–36.98)	0.203	27.75 (22.32–41.35)	0.141
>1,000	51	16.02 (9.65–36.78)		24.91 (20.71–35.96)	

*Mann-Whitney-Wilcoxon test was used.*

*CEPP, collection efficiency of plateletpheresis; IQR, interquartile range*.

### Factors Affecting the Collection Efficiency of Plateletpheresis and PLT Reduction Rate

**Figure 2:**
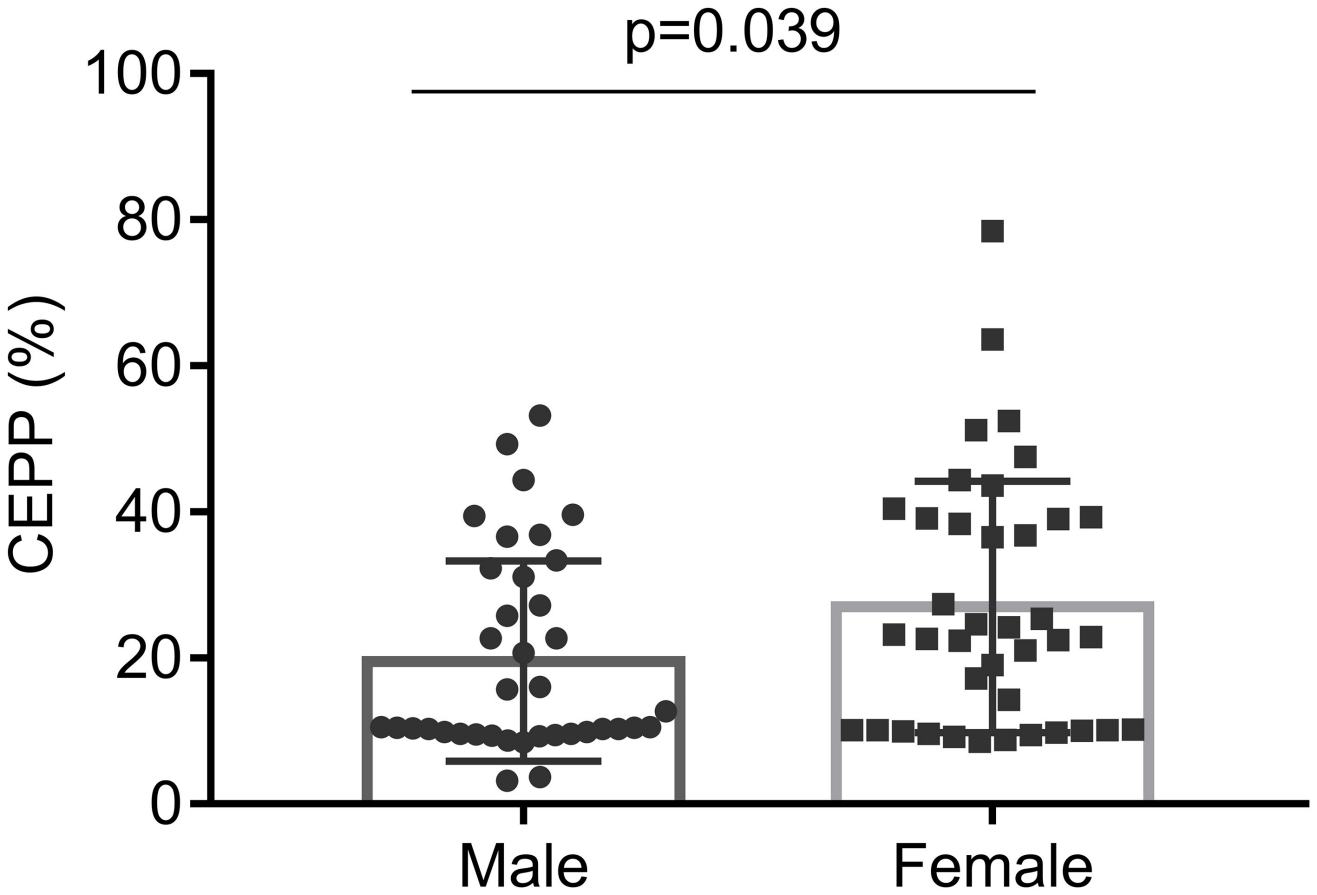
The comparison of CEPP between male and female.

**Figure 3:**
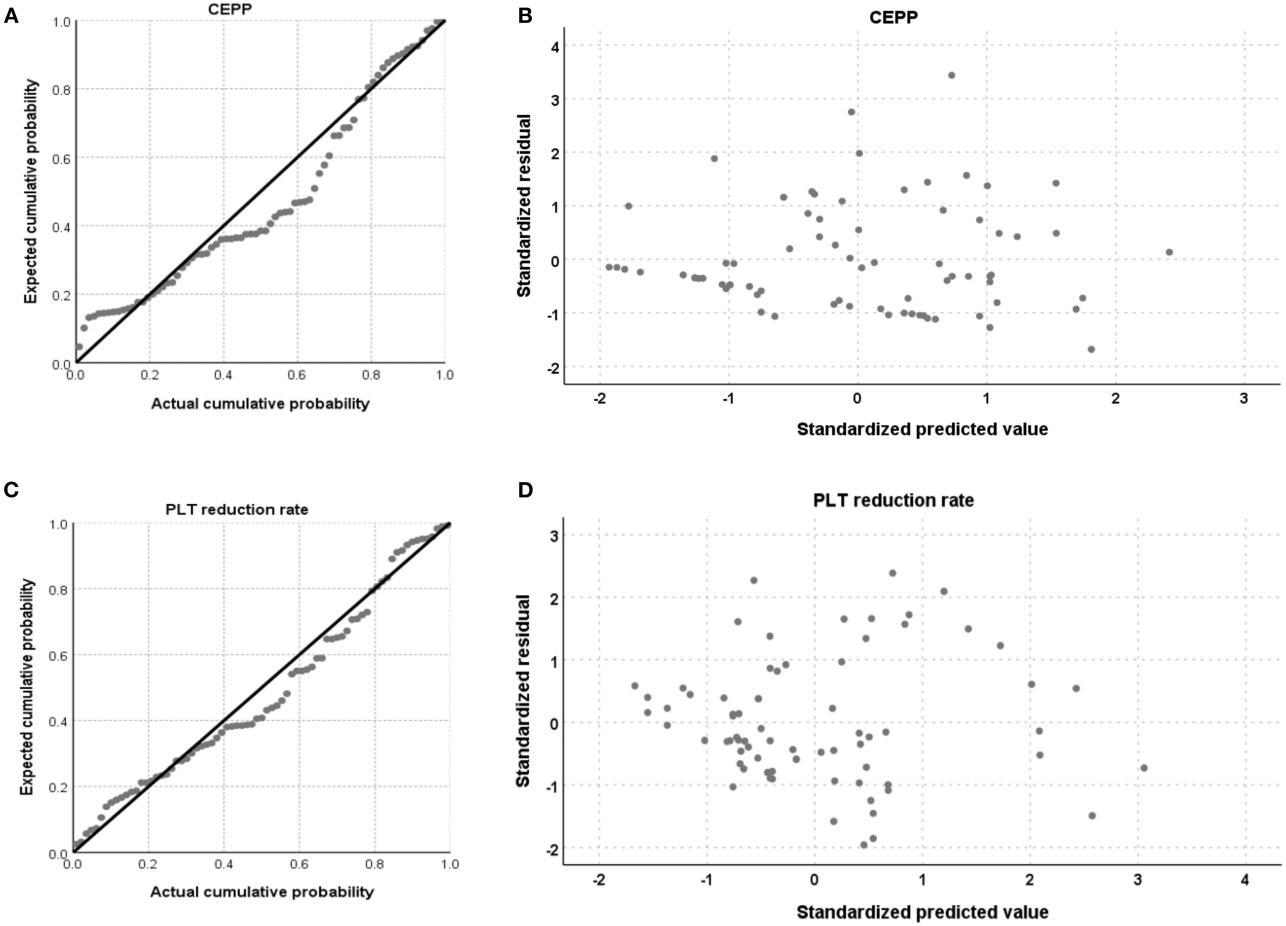
The model of P-P diagram and scatter of residual. The P-P diagram of CEPP **(A)**, and residual scatter of CEPP **(B)**, the P-P diagram of PLT reduction rate **(C)**, and residual scatter of PLT reduction rate **(D)**.

Pre-apheresis factors affecting the CEPP and PLT reduction rate are shown in Tables 4, 5. The CEPP was positively associated with HGB levels before plateletpheresis. Female patients achieved better CEPP than male patients, with an median CEPP of 10.54% in males and 22.86% females (Figure 2). The BVP had no influence on CEPP. Female sex (*P* = 0.022), HCT (*P* = 0.001), and blood volume (*P* = 0.015) were associated with PLT reduction rate. The model P-P diagram and scatter of residuals are shown in Figure 3.

**Table 4:** Factors affecting CEPP.

**Factor pre-apheresis**	**CEPP**	
	**B (95.0%CI)**	***p*-value**
Sex/Female	9.147 (2.389–16.445)	0.009
HGB	0.179 (0.043–0.314)	0.010
RBC	…	0.265
WBC	…	0.937
PLT	…	0.669
HCT	…	0.449
Blood volume	…	0.121

*CEPP, collection efficiency of plateletpheresis*.

**Table 5:** Factors affecting PLT reduction rate.

**Factor pre-apheresis**	**PLT reduction rate**	
	**B (95.0%CI)**	***p*-value**
Sex/Female	5.282 (0.776–9.788)	0.022
HGB	…	0.492
RBC	…	0.407
WBC	…	0.960
PLT	…	0.695
HCT	0.667 (0.296–1.038)	0.001
Blood volume	0.002 (0.000–0.004)	0.015

## Discussion

In primary thrombocytosis, such as in MPN, including ET, PV, CML, pre-fibrotic PMF, myelodysplastic syndrome, and rarely, AML, the platelet function is abnormal and thus, thrombocytosis is associated with thrombohemorrhagic events. The risk of bleeding increases significantly when the platelet count is >1,000 × 10^9^/L (9). Furthermore, too many PLT can result in several clinical symptoms, especially when platelets levels are >800 × 10^9^/L. Symptoms are predominantly vasomotor and may include headaches, dizziness, blurring of vision, syncope and erythromelalgia (24, 26). What's worse, PLT count outside of the normal range during follow-up was associated with an immediate risk of major hemorrhage (23). PLT activation and concomitant degranulation enables platelets to engage with leukocytes *via* soluble factors and physical interaction facilitated by a variety of receptors (32). Increased platelet-leukocyte aggregates (PLAs) within the circulation and/or locally at the site of inflammation represent markers of many thrombo-inflammatory diseases, such as cardiovascular diseases, acute lung injury, renal and cerebral inflammation (22). Massive release of several proinflammatory cytokines, especially interleukins 1, 6 and 8 and tumor necrosis factor-alpha, may occur after formation of PLAs, and these mediators can further contribute to increase sterile inflammation (25).

Thrombocytapheresis produces benefits of removing large numbers of platelets from the blood circulation and thus preventing morbidities (29, 33). Herein, we provide evidence that therapeutic plateletpheresis is an effective method of removing PLTs in patients with thrombocytosis. In this study, patients with PLT counts above 800 × 10^9^/L were enrolled and divided into two groups according to PLT counts. No significant differences were observed between PLT count with 800–1,000 × 10^9^/L and >1,000 × 10^9^/L.

CEPP was an indicator to compare the efficiency of different apheresis systems, and the higher CEPP means less processed volume to reach the same PLT yield (31). We concluded that the HGB level before apheresis significantly influenced the CEPP. Lower HGB is associated with relatively a higher proportion of plasma in the bloodstream, which allows the operator to reduce the volume collected in each cycle, and therefore the CEPP decreases. Therefore, the CEPP may be improved when the patients exhibit higher HGB. In addition, female patients had higher CEPP than male patients in this cohort, likely due to the lower blood volume in women. Therefore, we suggest to perform therapeutic plateletpheresis for MPN patients with PLT counts above 800 × 10^9^/L and high HGB, especially for women. This indicates that female patients with high HGB are more suitable for bridging therapy with plateletpheresis in thrombocytosis.

The mean platelet volume (MPV) is an important variable and is associated with the pathophysiological characteristics of various types of diseases, previous studies have found that platelet volume may be associated with a variety of prothrombotic and proinflammatory diseases (34, 35). In our data, only one patient presented MPV values lower than normal levels after plateletpheresis, and further analysis concluded there was no correlation between MPV pre-apheresis and CEPP. With regard to HCT, previous studies have reported significant effects on the efficiency of PLT collection, namely, an increase in the HCT significantly decreased the efficiency of platelet collection (36). Reinhart et al. recommended a transfusion threshold of 20–24% HCT in stable, hospitalized patients (37), and low HCT was suggested to reduce vascular thrombosis, cardiovascular disease, and other complications (38, 39). Braekkan et al. (39) found that HCT and related hematological variables were risk factors for venous thromboembolism. Jin et al. (38) indicated that elevated HCT levels may be positively associated with cardiovascular risk factors. The HCT did not significantly influence the CEPP in our data. Thus, different cut-off values may lead to different conclusions. Nonetheless, in our study, HCT values were associated with the PLT reduction rate.

Therapeutic plateletpheresis is also safe. As shown in Table 2, after apheresis, levels of RBC and HGB showed no significant differences before and after apheresis. The indicators of hepatorenal function significantly reduced to normal levels, and electrolytes exhibited no significant changes before and after apheresis, which suggested that plateletpheresis exhibited little adverse effects or toxicity on organs and did not influence the body's overall homoeostasis compared to chemotherapy. Plateletpheresis involves the separation of abnormal blood cells from the body, while chemotherapy can lead to the death of normal and malignant cells including immune cells, with additional complications. In recent years, different devices have been developed to perform plateletpheresis, such as the Fenwal Amicus Blood Cell Separator (40) and the Haemonetics MCS Cell Separator (30). Zhou et al. reported that no severe adverse reactions was detected during platelet apheresis on MCS+ (41). Similar to previous studies, no serious side effects occurred when the PLT count significantly decreased with a median PLT reduction rate of 25.34%. Importantly, symptoms of dizziness, headache, somnolence, and stupor were relieved significantly after plateletpheresis.

Changes in coagulation factors were also analyzed, and indicated that apheresis had no effects on coagulation functions. PLTs play an important role in blood coagulation. Rapid cytoreduction is believed to ameliorate prothrombotic factors associated with dysfunctional platelets (9). However, treatment with anticoagulants also influenced platelet counts during plateletpheresis, and platelet levels were not related to coagulation parameters. Fibrinogen and anti-thrombin (AT) decrease significantly with prolonged PT and activated partial thromboplastin time (APTT), but fall within an acceptable range (42) or remain within normal limits during double plateletpheresis (43).

According to the ASFA guidelines on the use of thrombocytosis, which were established to assist the requesting and/or apheresis physicians in evaluating the utility of apheresis as a treatment modality, therapeutic apheresis has Grade 2C recommendations for symptomatic, prophylactic, or secondary causes (9). Therapeutic apheresis has been suggested to prevent recurrence or to treat acute thromboembolism or hemorrhage, but we believe that it is effective in relieving symptoms, and thrombocytosis presenting serious symptoms should be treated with therapeutic apheresis promptly. For plateletpheresis, the leuko-depleted platelet collection is very important for donor PLT collection and to prepare PLT concentrates for transfusion, but not for therapeutic apheresis (44).

Our study presents some limitations, such as the small number of patients. Therapeutic apheresis can reduce the risk of thrombosis or stroke in the short-term, but long-term treatment also needs to be accompanied by chemotherapeutic drugs and other measures.

Taken together, our results show that therapeutic plateletpheresis was very effective in reducing PLT counts with acceptable efficiency. We propose that it is reasonable to define the PLT threshold for plateletpheresis as 800 × 10^9^/L when patients are complicated by clinical symptoms such as dizziness, headache, somnolence, and stupor. Plateletpheresis is effective in removing PLT especially with high HGB in female patients. In conclusion, our findings provide implications for future research with regard to the resolution of symptoms and therapeutic plateletpheresis. Our findings will help to direct hematology/oncology professionals and apheresis physicians to better understand the efficacy and limitations of therapeutic apheresis and provide information for more rational clinical decision-making for patients with thrombocytosis.

## Data Availability Statement

The raw data supporting the conclusions of this article will be made available by the authors, without undue reservation.

## Ethics Statement

Ethical review and approval was not required for the study on human participants in accordance with the local legislation and institutional requirements. Written informed consent for participation was not required for this study in accordance with the national legislation and the institutional requirements.

## Author Contributions

HJ, YS, and YJ analyzed the data, drew pictures, and wrote the manuscript. GY collected and analyzed the data. DL, JL, and CW performed the operation. LD and XT collected the data. SG, FG, and FZ designed the project and provided professional guidance. FZ revised the manuscript. All authors contributed to the article and approved the submitted version.

## Funding

This article was supported by the National Natural Science Foundation of China (NSFC) program (No. 81770179) and Hong Kong Scholars Program (No. XJ2018060).

## Conflict of Interest

The authors declare that the research was conducted in the absence of any commercial or financial relationships that could be construed as a potential conflict of interest.

## Publisher's Note

All claims expressed in this article are solely those of the authors and do not necessarily represent those of their affiliated organizations, or those of the publisher, the editors and the reviewers. Any product that may be evaluated in this article, or claim that may be made by its manufacturer, is not guaranteed or endorsed by the publisher.
